# Low-Temperature Ferromagnetic Order in a Two-Level Layered Co^2+^ Material

**DOI:** 10.1021/acs.chemmater.4c00596

**Published:** 2024-08-09

**Authors:** Patrick W. Doheny, Gavin B. G. Stenning, Adam Brookfield, Fabio Orlandi, David Collison, Pascal Manuel, Sam T. Carr, Paul J. Saines

**Affiliations:** † School of Chemistry and Forensic Science, Ingram Building, University of Kent, Canterbury CT2 7NH, U.K.; ‡ ISIS Neutron and Muon Source, Rutherford Appleton Laboratory, Chilton, Didcot OX11 0QX, U.K.; § Department of Chemistry and Photon Science Institute, EPSRC National Research Facility for Electron Paramagnetic Resonance Spectroscopy, The University of Manchester, Oxford Road, Manchester M13 9PL, U.K.; ∥ School of Physics and Astronomy, Ingram Building, University of Kent, Canterbury CT2 7NH, U.K.

## Abstract

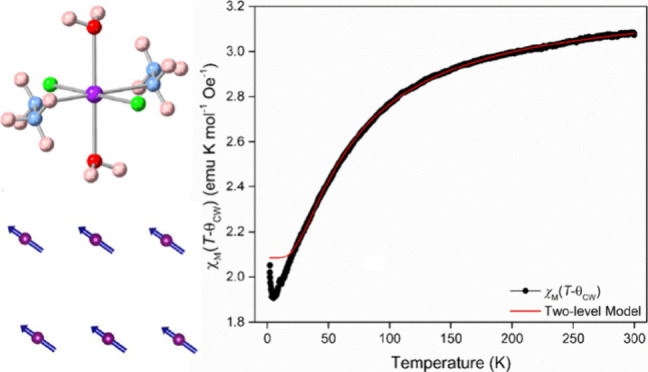

The magnetic properties of a 2D layered material consisting of high-spin Co^2+^ complexes, [Co(NH_3_NH_2_)_2_(H_2_O)_2_Cl_2_]Cl_2_ (**CoHyd**_**2**_**Cl**_**4**_), have been extensively characterized using electron paramagnetic resonance, magnetic susceptibility, and low-temperature heat capacity measurements. Electron paramagnetic resonance spectroscopy studies suggest that below 50 K, the *J* = 3/2 orbital triplet state of Co is gradually depopulated in favor of the *J* = 1/2 spin state, which is dominant below 20 K. In light of this, the magnetic susceptibility has been fitted with a two-level model, indicating that the interactions in this material are much weaker than previously thought. This two-level model is unable to fit the data at low temperatures and, combined with electron paramagnetic resonance spectroscopy, suggests that ferromagnetic interactions between Co^2+^ cations in the *J* = 1/2 state become significant approaching 2 K. Heat capacity measurements suggest the emergence of a long-range ordered state below 246 mK, which neutron diffraction confirms to be ferromagnetic.

## Introduction

Low-dimensional magnetic materials have attracted sustained interest due to the exotic and often unconventional magnetic properties that arise within them.^1^ This includes cases of competing magnetic interactions that cannot be mutually satisfied, so-called geometrically frustrated structures, examples of which include the 2D kagome,^2−4^ triangular lattices,^5−7^ and 3D pyrochlores.^8−10^ Although many of the materials that have been examined for magnetic frustration have been *S* = 1/2 systems, such as those based on Cu^2+^, other magnetic systems incorporate the Co^2+^ ion, which commonly adopts a high-spin *S* = 3/2 state.^11−15^ The greater single ion anisotropy of Co^2+^, which arises from its orbital angular momentum not being fully quenched in the commonly adopted octahedral environment, often leads to its spins exhibiting Ising-like behavior rather than the Heisenberg behavior adopted by Cu^2+^, modifying the frustration within them. Thus, incorporating Co^2+^ ions into systems with well-defined magnetic dimensionality is of particular interest for studying magnetic materials.

As a result of high-spin octahedral Co^2+^ retaining significant orbital angular momentum, *L*, its lowest energy state is split by first-order spin–orbit coupling into three separate energy levels, with *J* = 1/2, *J* = 3/2, and *J* = 5/2 in order of increasing energy.^16^ At ambient temperature, only the *J* = 1/2 and *J* = 3/2 states are usually occupied due to the *J* = 5/2 level being too high in energy. On cooling, the *J* = 3/2 state is progressively depopulated in favor of the *J* = 1/2 state, enabling data obtained at low temperatures to be modeled as an *S*_eff_ = 1/2 state.^16^ The significant orbital angular momentum of the *J* = 1/2 state enables it to exhibit strong anisotropic magnetic interactions required for the bond-dependent Ising couplings associated with the Kitaev model, a route toward magnetically frustrated spin liquids.^17,18^ The depopulation of the higher energy *J* = 3/2 spin–orbit coupled state of Co^2+^ in favor of its low-spin *J* = 1/2 state at low temperatures does, however, cause potential complications in the understanding of such materials.^16,19−21^ In particular, extracting the strength of the magnetic interactions from magnetic susceptibility measurements can be difficult, as the increase in population of the *J* = 1/2 state can lead to trends in bulk magnetic susceptibility measurements that can be misinterpreted as significant antiferromagnetic interactions in the material.

The most well-studied Co^2+^ magnets are purely inorganic materials, which typically adopt close-packed structures that lack sufficient spacing between their low-dimensional units to magnetically isolate them. New magnetic materials containing both organic and inorganic building blocks provide an alternative route to realizing well-isolated, low-dimensional units because of the structures they adopt to accommodate their non-spherical molecular components. Many of these materials are only studied by bulk property measurements with detailed studies of these materials with other techniques relatively limited.^22^ While much of the interest in organic–inorganic magnets focuses on coordination polymers and dense metal–organic frameworks, in which the organic building blocks link magnetic ions,^22^ an alternate approach is to use the organic molecules as spacers between inorganic units. Among such materials, complexes in which the magnetic coupling occurs via halide close contacts have drawn particular attention, which have been shown to be hosts to spin chains,^23−25^ sheets,^26−28^ and ladders.^29−31^ Much of the work in this area is based on A_2_MX_4_ tetrahalometallates, in which the magnetic ion is four-coordinate, and the A cation is a relatively bulky ammonium cation that acts purely as a spacer unit.^29,32,33^ Recently, we have, however, reported possible magnetic frustration in the octahedral Co^2+^ complex [Co(NH_3_NH_2_)_2_(H_2_O)_2_Cl_2_]Cl_2_ (**CoHyd**_**2**_**Cl**_**4**_, where Hyd is hydrazinium), in which the smaller amines are coordinated to a single Co^2+^.^34^ The shortest Cl···Cl contacts between magnetic cations comprise an edge-sharing rhomboidal lattice, with the next shortest Cl···Cl contacts forming chains such that together an edge-sharing triangular lattice emerges. The behavior of this material below 2 K, including whether at some low temperature the material magnetically orders, was not examined.

In this work, we explore the magnetic behavior of **CoHyd**_**2**_**Cl**_**4**_ in more detail. Electron paramagnetic resonance (EPR) spectroscopy studies indicate the gradual depopulation of the *J* = 3/2 orbital triplet state in favor of the *J* = 1/2 state, which appears to be exclusively populated below 20 K. Fitting the magnetic susceptibility measurements with a two-level model indicates that the magnetic interactions are significantly weaker than previously suggested and the material is unlikely to exhibit significant magnetic frustration. There is evidence of ferromagnetic interactions at low temperatures not accounted for by the two-level model. Heat capacity measurements indicate the onset of long-range magnetic order at 246 mK, which neutron diffraction indicates is to a ferromagnetic state. The dominance of the *J* = 1/2 state in the ordered magnetic phase is broadly consistent with the entropy associated with the magnetic phase transition and ordered magnetic moment refined from fits to the neutron diffraction patterns.

### Synthesis and Structure

**Figure 1:**
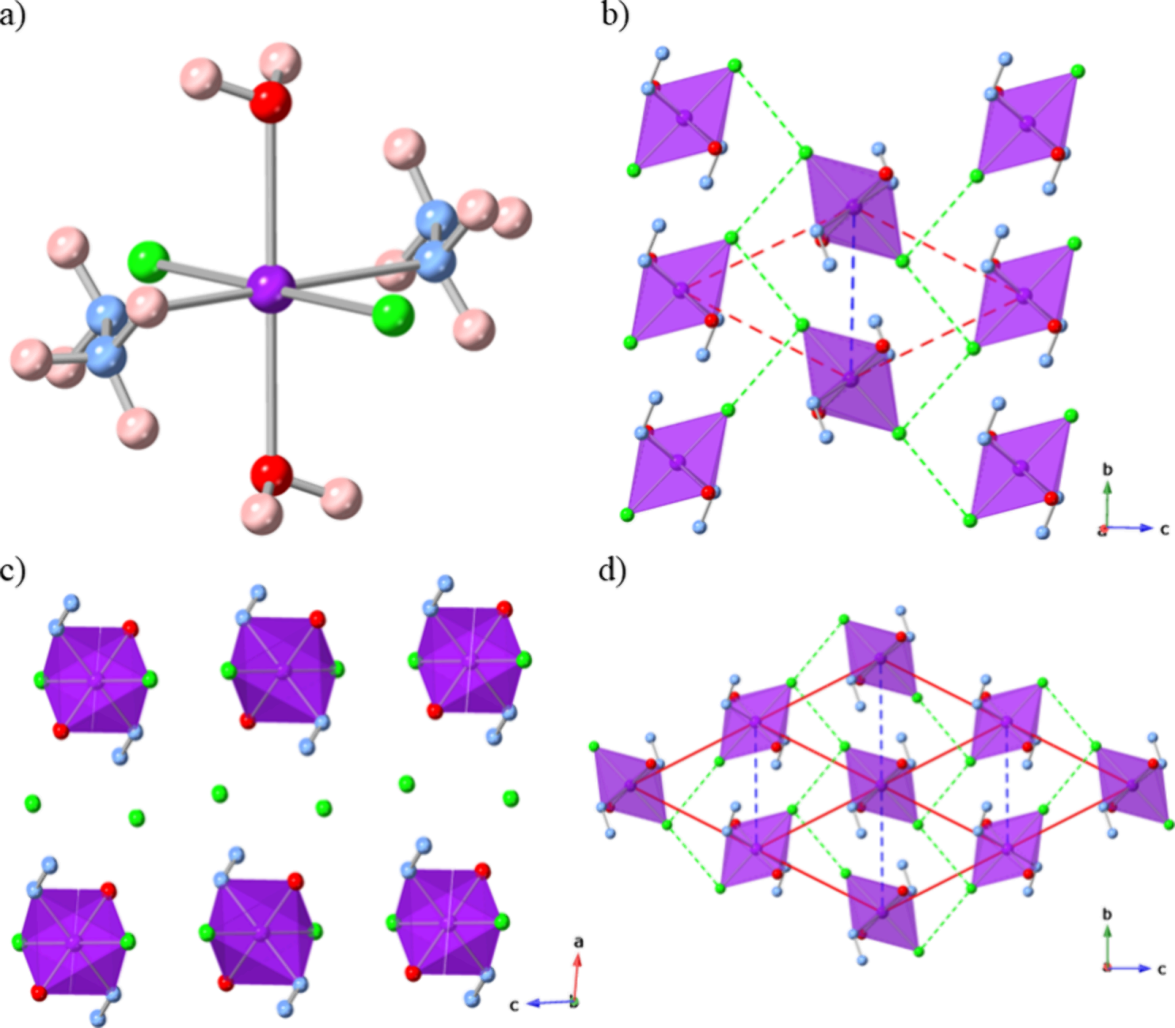
Crystal structure of **CoHyd**_**2**_**Cl**_**4**_ showing the (a) octahedral complex, (b) 2D layers formed by the complexes in the *bc*-plane, (c) individual **CoHyd**_**2**_**Cl**_**4**_ layers separated by chloride anions, and (d) extended *bc*-plane showing the rhomboidal structure. Nearest-neighbor Cl···Cl contacts are indicated by dashed green lines, while the dashed red lines indicate the Co centers forming one rhomboid and the dashed blue lines show the Co nearest neighbors. The hydrogen atoms in parts (b) and (c) have been omitted for clarity with the Co octahedra shown in purple. Atom labeling: Co = purple, Cl = green, O = red, *N* = blue, and H = pink.

The **CoHyd**_**2**_**Cl**_**4**_ complex crystallizes in a monoclinic *P*2_1_/*c* structure characterized by octahedral Co atoms with two hydrazinium cations, two water molecules, and two chloride anions as ligands with like ligands coordinating in a *trans* fashion to the Co atoms (Figure 1a). The bulk structure consists of alternating layers of **CoHyd**_**2**_**Cl**_**4**_ complexes in a rhomboidal-like arrangement along the *bc*-plane (Figure 1b,d) separated by undulating layers of Cl^–^ anions stacked along the crystallographic *a*-axis (Figure 1c).

Neighboring complexes in the *bc*-plane are linked through zigzag chains of nearest-neighbor Cl···Cl contacts of 3.682(11) Å to give a magnetic superexchange distance of 8.466(14) Å and an associated torsion angle of 138(5)°. Collectively, these nearest-neighbor contacts lead to a rhomboidal lattice. There are separate 1D Co–Cl···Cl–Co chains along the *c*-axis that include the next-nearest intralayer Cl···Cl contact of 3.947(15) Å and a superexchange distance of 8.73(2) Å with an associated torsion angle of 180°; these divide the rhomboids to form a triangular lattice. The shortest interlayer Cl···Cl contact was determined to be slightly longer at 3.816(10) Å with the other interlayer Cl···Cl contact required, in concert, to connect Co cations in neighboring layers at 4.274(10) Å. Given that any coupling between Co^2+^ centers must be mediated by Cl atoms, any significant magnetic coupling is likely to be between intralayer complexes rather than interlayer complexes where the much larger Co–Cl···Cl···Cl–Co pathways would diminish any magnetic interactions.

Bulk polycrystalline samples of **CoHyd**_**2**_**Cl**_**4**_ were synthesized by mixing a solution of CoCl_2_·6H_2_O in MeOH:H_2_O with a solution of hydrazinium chloride in MeOH:H_2_O in a 1:2 molar ratio. The resulting solution was then transferred to a Schlenk flask and evaporated under a positive pressure of N_2_ gas to yield a dark pink powder of the target complex. Samples for neutron powder diffraction were obtained using the same method, with the substitution of MeOH for MeOD and water for D_2_O to yield the deuterated phase. The phase purity of the obtained solid was confirmed using powder X-ray diffraction analysis (Figure S1), which was consistent with the previously reported structure.^34^

### EPR Spectroscopy

EPR spectroscopy was carried out to establish the spin state of the system at a low temperature. This was initially performed at X-band frequencies over a 2.25–140 K temperature range (Figure S2), which were characterized by broad, isotropic spectra with a low signal-to-noise ratio at higher temperatures, consistent with the fast spin relaxation time of Co^2+^ ions.^16,35,36^ Below 60 K, the formation of an axial signal, where *g*_*x*,*y*_ > *g*_*z*_, was observed, with this anisotropic component of the signal increasing in intensity with decreasing temperature. A consistent shift to lower fields, and hence, a larger *g*-value was also observed for the *g*_*x*,*y*_ component of the signal with decreasing temperature. Despite the obvious *g*-anisotropy of the signal, the broadness of the spectra prevented this anisotropy from being resolved, and so EPR data at Q-band frequencies were collected to elucidate this further.

**Figure 2:**
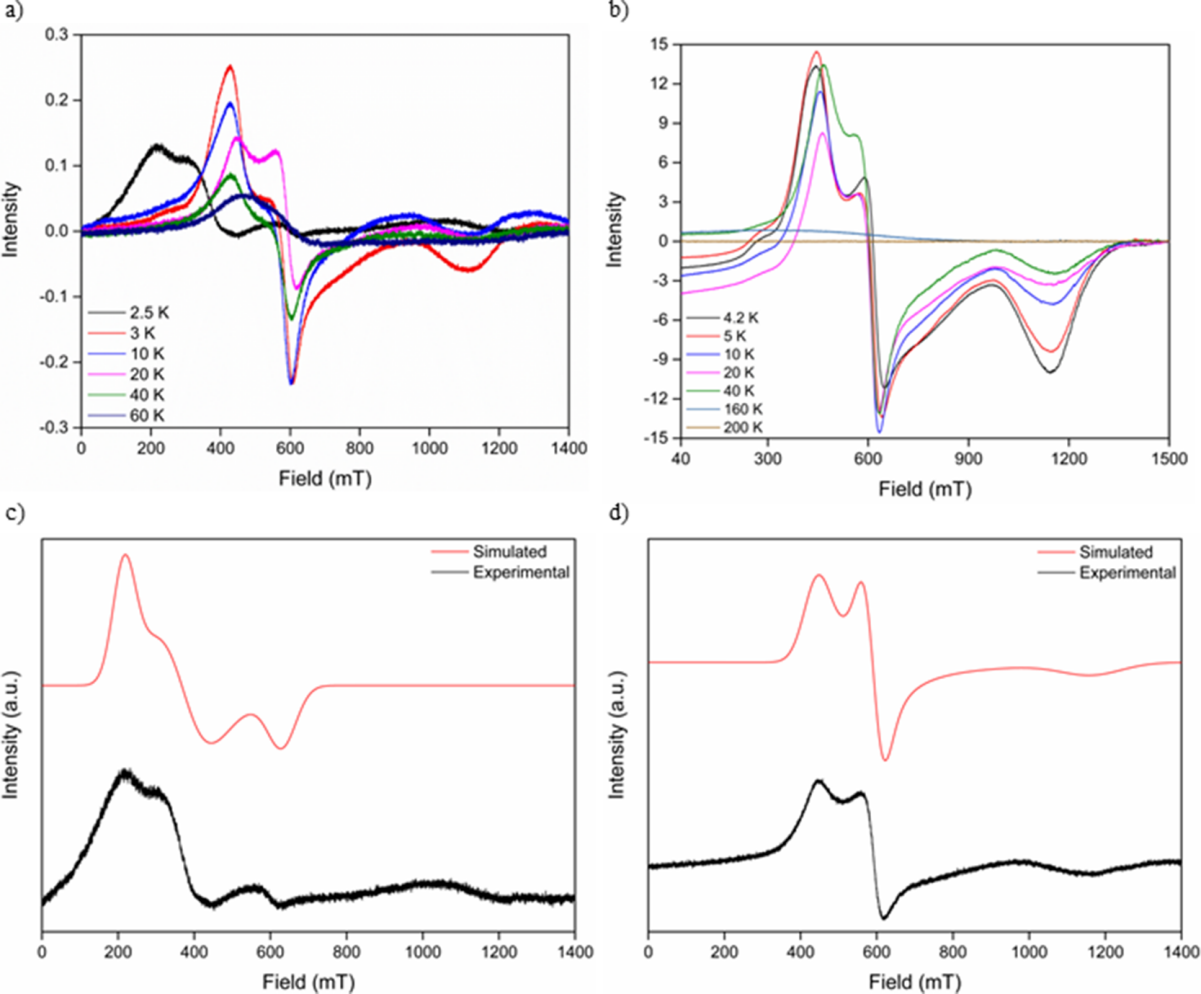
Variable-temperature Q-band EPR spectroscopy of the **CoHyd**_**2**_**Cl**_**4**_ material collected over (a) 2.5–60 K and (b) 4.2–200 K temperature ranges. Experimental vs simulated spectra of the **CoHyd**_**2**_**Cl**_**4**_ EPR signal at (c) 2.5 K using a simulated *g*-matrix of *g*_*x*_ = 3.851, *g*_*y*_ = 6.436, and *g*_*z*_ = 11.091 and (d) 20 K using a simulated *g*-matrix of *g*_*x*_ = 2.069, *g*_*y*_ = 4.097, and *g*_*z*_ = 5.458.

Variable-temperature Q-band EPR data were collected over a 2.5–60 K (Figure 2a) and 4.2–200 K (Figure 2b) temperature range (see Figures S3 and S4 for the full temperature range), which was successful in resolving the *g*-anisotropy indicated by the X-band data. The Q-band spectra at low temperatures were characterized by a rhombic (*g*_*x*_ ≠ *g*_*y*_ ≠ *g*_*z*_) *g*-matrix below 80 K with the spectra becoming progressively broader and a decrease in the signal-to-noise ratio above this temperature. Consistent with the X-band data, a shift to lower fields (and hence higher *g*) was observed for all three components of the *g*-matrix with decreasing temperature. This suggests that intermolecular interactions become significant at low temperatures as the thermal energy of the system decreases.

The low-temperature EPR spectra were simulated by treating the Co^2+^ ions as an *S*_eff_ = 1/2 state rather than a spin-only *S* = 3/2 high-spin state, reflecting the splitting of the octahedral Co^2+^ energy levels due to spin–orbit coupling.^16^ Good resemblance is achieved between the model and experimental data, indicating that the *J* = 1/2 level is exclusively populated at low temperatures (see Figure 2). Simulation of the Q-band data at 2.5 (Figure 2c) and 20 K (Figure 2d) highlighted the large shift in *g*-values, particularly *g*_*z*_, with respect to the system temperature where the increase in the *g*-values of all three components of the rhombic signal indicated the strengthening of intermolecular interactions between Co^2+^ ions. The shift to higher *g*-values (Table 1) was also observed in the 4.2–200 K data set; however, the absolute shifts are much smaller, which likely arises from the fact that the base temperature of 4.2 K is much higher than 2.5 K, compared to the strength of the interactions between *J* = 1/2 Co^2+^ cations in **CoHyd**_**2**_**Cl**_**4**_.

**Table 1:** Comparisons of **CoHyd**_**2**_**Cl**_**4**_*g*-Values Obtained from Simulations of Q-Band EPR Spectra Obtained at 2.5 and 20 K Using a Bruker SuperQ-FT Bridge and at 4.2 and 20 K Obtained Using a Bruker EMXPlus Platform

**temperature (K)**	***g*_*x*_**	***g*_*y*_**	***g*_*z*_**
2.5	3.851	6.436	11.091
20	2.069	4.097	5.458
4.2	2.176	4.055	5.680
20	2.169	4.188	5.471

### Magnetic Susceptibility and Heat Capacity Analysis

The magnetic susceptibility of the bulk material was examined by using SQUID magnetometry over a 2–300 K temperature range under an applied field of 1000 Oe. Neither the field-cooled nor zero-field-cooled susceptibility (Figure S5) showed any indication of magnetic ordering down to 2 K. The inverse susceptibility curve (Figure S6) was initially fitted using the Curie–Weiss law over a 50–300 K temperature range resulting in a Weiss temperature, θ_CW_, of −17.88(2) K (see Table 1), which is very similar to that from the previous study of **CoHyd**_**2**_**Cl**_**4**_, from which a value of −18.75 K was obtained.^34^ The effective magnetic moment, *μ*_eff_, was calculated as 5.1095(4) *μ*_B_, substantially higher than the predicted spin-only value (3.87 *μ*_B_), which was attributed to significant spin–orbit coupling contributions to the magnetic moment.^37,38^ We note that the value determined for *μ*_eff_ is close to the upper limit typically measured for high-spin Co^2+^ materials with completely orbital unquenched angular momentum.^39,40^

**Figure 3:**
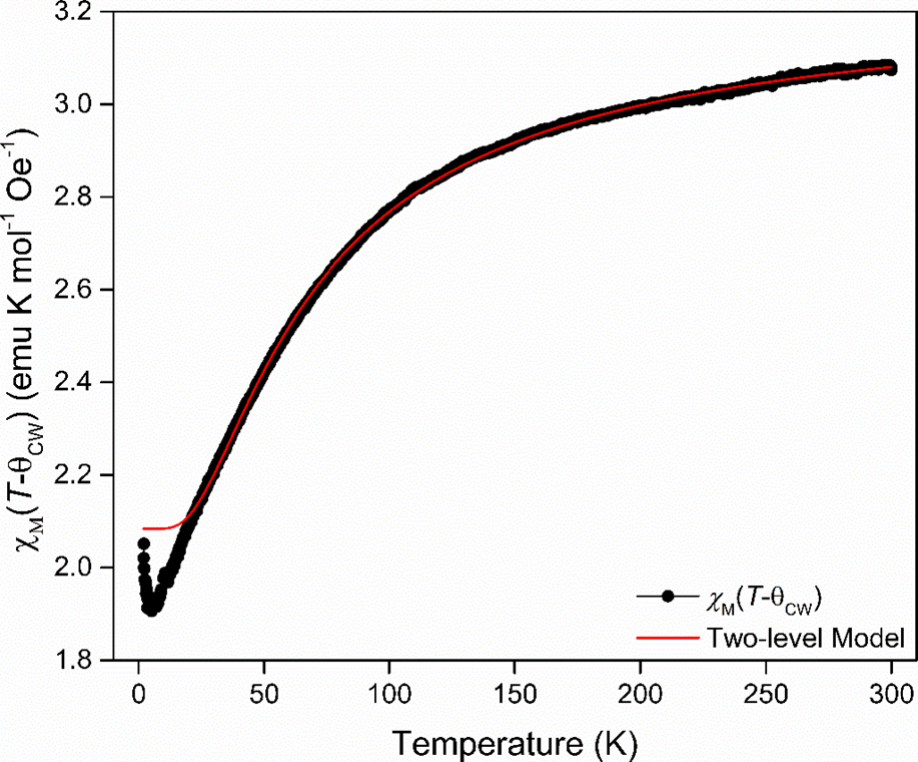
Two-level fit to the evolution of χ_M_ plotted as χ_M_(*T* – θ_CW_) as a function of temperature.

It should be noted that the inverse susceptibility was not fitted over the entire 2–300 K temperature range as deviations from linearity were observed below 50 K. While such deviations are often associated with antiferromagnetism, a similar signal can be caused by the depopulation of the *J* = 3/2 orbital triplet state in favor of the lower energy *J* = 1/2 state. We therefore fitted the magnetic susceptibility data using the two-level model:^41,42^
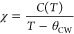
where
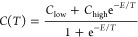
where *C*_low_ and *C*_high_ are the Curie constants of the low and high energy states and *E* is the energy gap between them in K. This model was fitted to 1/χ across the full data range giving values of *C*_low_ = 2.1 emu K mol^–1^ Oe^–1^, *C*_high_ = 4.4 emu K mol^–1^ Oe^–1^, *E* = 88 K, and θ_CW_ = −0.02 K (see Figure 3 for the fit replotted as *χ*_M_(*T* – θ_CW_) versus temperature). This gives effective magnetic moments of 4.1 *μ*_B_ and 5.9 *μ*_B_ for the low and high energy states. Although the value for the low energy state of 4.1 *μ*_B_ initially appears high for an *S*_eff_ = 1/2 system, it is relatively close to the value of 3.61 *μ*_B_ calculated for the effective magnetic moment taking the values from the EPR fit at 20 K. This is consistent with the very large contribution from the orbital angular momentum observed in other Co^2+^*J* = 1/2 states, which also exhibit significant magnetic anisotropy.^43,44^ The θ_CW_ value is much lower than that obtained from the Curie–Weiss fit suggesting that the magnetic interactions in this material are much weaker than previously suggested.^34^

As can be seen most clearly in Figure 3, the two-level model is unable to fit the observed trend in magnetic susceptibility below 20 K. This is primarily due to the increase observed near 2 K, which is also observed in a plot of χ_M_*T* data versus temperature (Figure S7). This suggests that ferromagnetic interactions become significant near 2 K, consistent with the very large shift in *g*-values observed in the EPR data. Such ferromagnetic interactions would not be predicted from the θ_CW_ obtained from the two-level model, which suggests that either this is somewhat correlated with *E* or these interactions only emerge at low temperatures.

**Figure 4:**
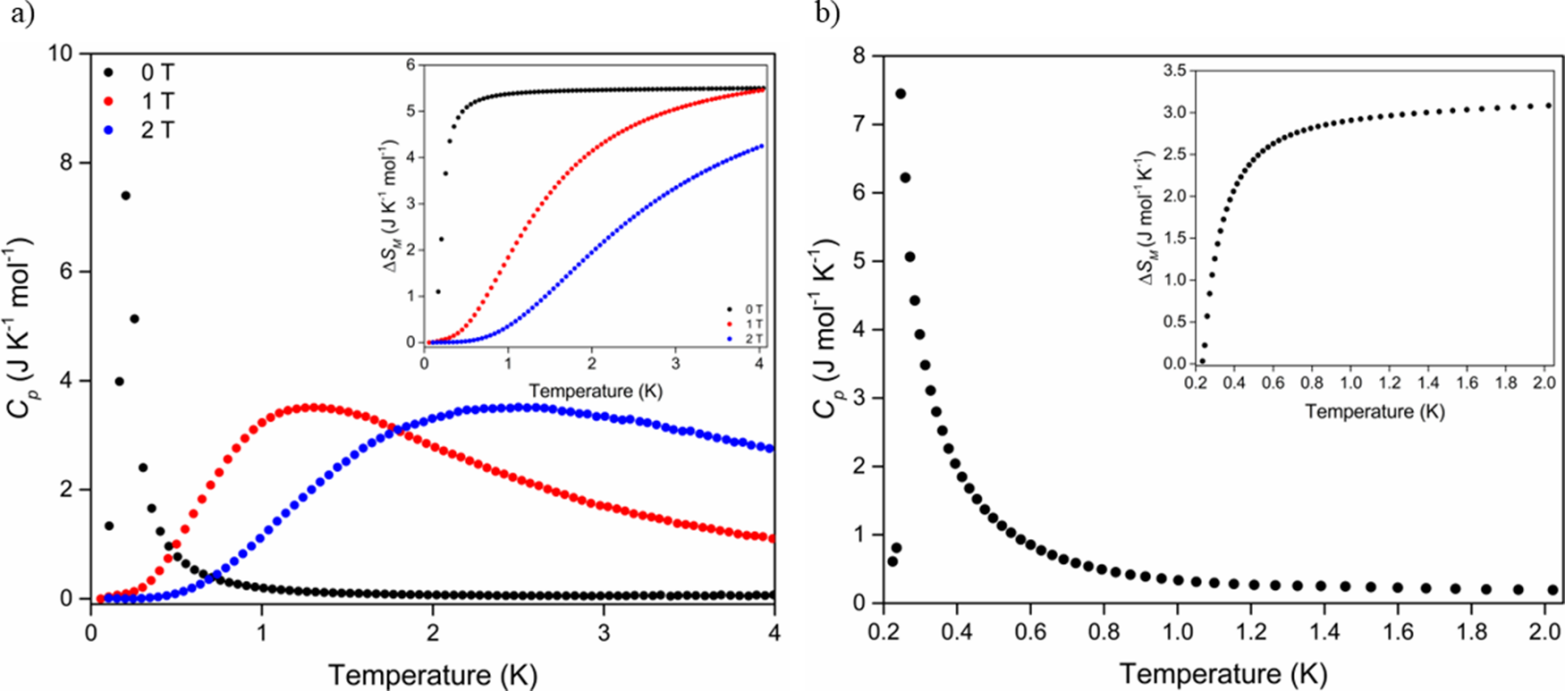
(a) Low-temperature heat capacity data of **CoHyd**_**2**_**Cl**_**4**_ measured between 0.1 and 4.0 K under applied fields of 0–2 T showing magnetic ordering at 246 mK and 0 T. (b) Heat capacity measured between 0.224 and 2.2 K under 0 T using finer temperature points to better capture the magnetic transition. Insets: the changes in magnetic entropy associated with the ordering transitions.

In order to determine the ordering temperature of **CoHyd**_**2**_**Cl**_**4**_, low-temperature heat capacity measurements of the sample were carried out using a ^3^He/^4^He dilution refrigerator that allowed measurements below 2 K. In a zero applied field (Figure 4), the heat capacity rapidly increased below 0.6 K to reach a maximum at 246 mK. Measurements under applied magnetic fields show that the peak at the transition temperature is suppressed and broadens to higher temperatures, consistent with this feature being caused by magnetic order. From the *C*_*p*_/*T* data (Figures S8 and S9), the magnetic entropy change of **CoHyd**_**2**_**Cl**_**4**_ at this ordering transition was also calculated to yield a magnetic entropy change of 5.05 J mol^–1^ K^–1^ at 0 T, with a similar entropy change extracted from the broader feature observed under a 1 T field change of 5.03 J mol^–1^ K^–1^. This value is close to that of the *S*_eff_ = 1/2, Co^2+^ configuration (5.76 J mol^–1^ K^–1^).

### Neutron Powder Diffraction and Magnetic Structure

**Figure 5:**
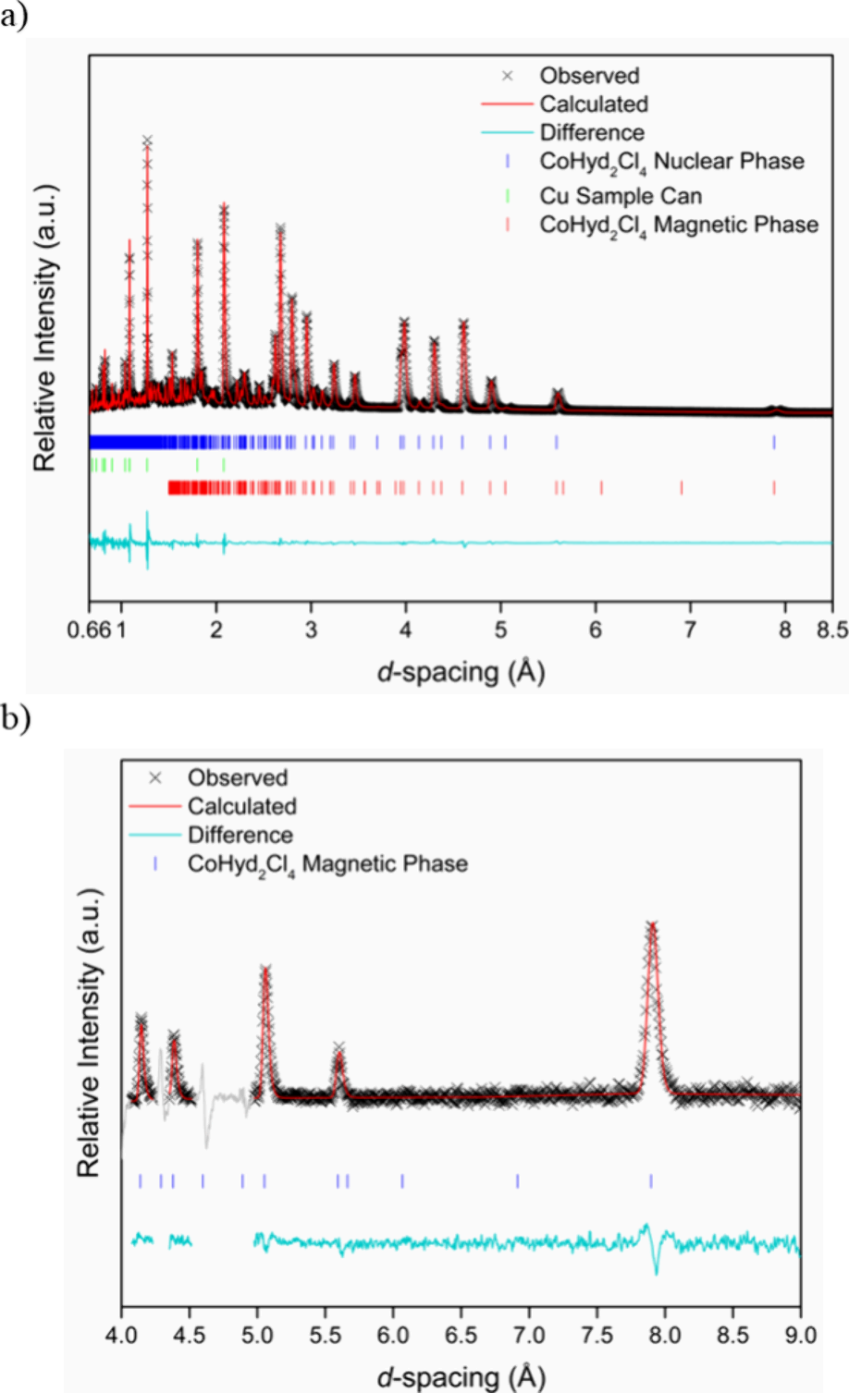
(a) Rietveld refinement profile of the **CoHyd**_**2**_**Cl**_**4**_ neutron powder diffraction data obtained at 87 mK from WISH detector banks 2 and 9, with an average 2θ of 58.33°, where *a* = 7.9605(4) Å, *b* = 5.6554(3) Å, *c* = 11.2819(6) Å, *β* = 97.926(3)°, and *V* = 503.06(5) Å^3^. *R*_p_ = 10.1% and *R*_wp_ = 4.68%. (b) Fits to the magnetic Rietveld refinement profile of the neutron powder diffraction data at 87 mK from which data collected at 20 K have been subtracted. The gray regions in these data were excluded from the refinement as they correspond to strong nuclear reflections.

In order to determine the magnetic structure of **CoHyd**_**2**_**Cl**_**4**_, neutron powder diffraction was carried out using the WISH beamline at the ISIS Neutron and Muon Source.^45^ Initial cooling of the sample to 2 K did not lead to the emergence of new peaks or increases in intensity, consistent with a lack of magnetic ordering, as indicated by the heat capacity data. Upon cooling to 87 mK, however, additional intensities due to magnetic Bragg scattering were observed to appear on top of the peaks corresponding to the nuclear phase. This indicated the magnetic phase adopted a **k**-vector of (0,0,0) and allowed the possible magnetic structures to be determined using the SARAh^46^ and ISODISTORT codes.^47,48^ The resulting magnetic structure was solved in the *P*2_1_′/*c′* magnetic space group with the corresponding irreducible representation (irrep) of mΓ_2_^+^, transformation matrix of [(1 0 0), (0 1 0), (0 0 1)], and origin of (0,0,0) with respect to the nuclear structure. A good fit was only obtained for the low-temperature neutron data via Rietveld refinement using a model employing modes associated with a collinear ferromagnetic arrangement of Co^2+^ spins (Figures 5a, S10 and Tables S1 and S2). The quality of the fit obtained with the model using modes associated with the mΓ_2_^+^ irrep was also checked against the difference neutron pattern at 87 mK, where the contributions to the Bragg peaks from the nuclear scattering at 20 K were removed. The difference pattern (Figure 5b) was fitted well using the collinear ferromagnetic structure, in contrast to the analogous antiferromagnetic structure model, enabled by modes associated with the alternate irrep mΓ_1_^+^, which did not reproduce the intensities of the magnetic Bragg peaks and was discarded (Figure S11).

**Figure 6:**
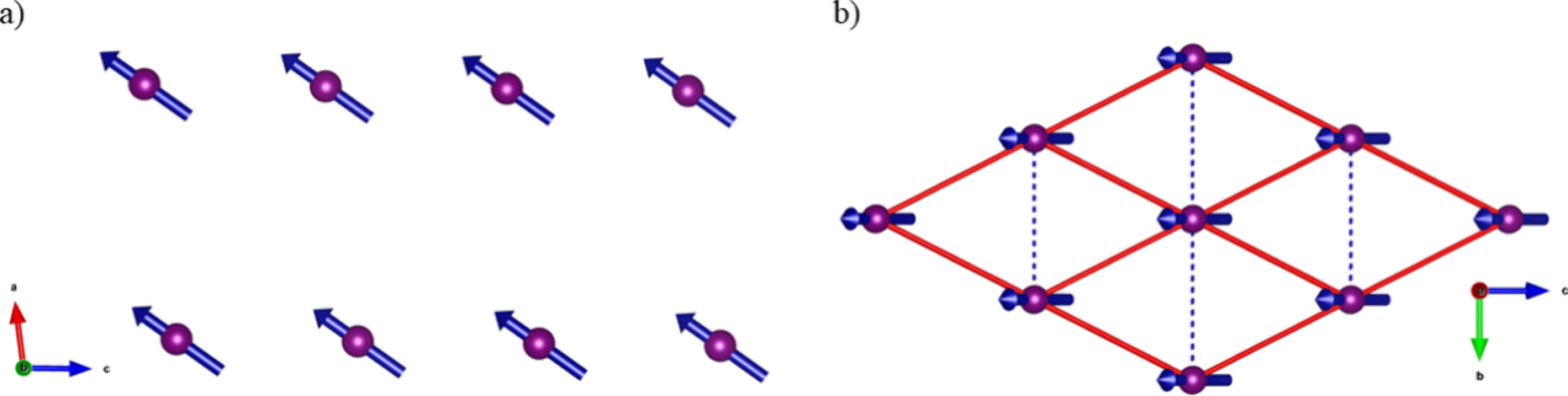
Magnetic structure of **CoHyd**_**2**_**Cl**_**4**_ showing (a) ferromagnetic ordering along the *ac*-plane and (b) the rhomboidal network formed from the Co^2+^ ions along the *bc*-plane. The red lines connect the next-nearest Co neighbors into a rhomboidal lattice, while the dashed blue lines show the Co nearest neighbors.

The magnetic structure (Figure 6a) was found to consist of a collinear arrangement of Co^2+^ moments orientated along the approximate [101̅] direction of the structure. When viewing the layers in the structure, the rhomboidal lattice (Figure 6b) associated with the shortest Cl···Cl contact is between next-nearest Co atoms in space, at 6.3105(5) Å, while the nearest-neighbor Co atoms are at a distance of 5.6560(6) Å through-space and are connected by the next-nearest Cl···Cl contact to complete a triangular lattice.

From the magnetic structure at 87 mK, a total refined magnetic moment of 1.69(2) *μ*_B_ was obtained per Co^2+^ atom (Figure S12). This increased slightly on initial heating suggesting that the sample may not quite have thermally equilibrated at the base temperature before progressively decreasing as the temperature increased before rapidly decreasing above 260 mK. This moment is relatively close to the value of 2.09 *μ*_B_ expected for an *S*_eff_ = 1/2 state given the *g*-value indicated by EPR measurements at 20 K, i.e., above the temperature that magnetic interactions become significant. That **CoHyd**_**2**_**Cl**_**4**_ adopts a *J* = 1/2 state at low temperatures suggests that related inorganic–organic materials with Cl···Cl may be suitable hosts for Kitaev physics although higher symmetry structures will likely be required to avoid the ferromagnetic state observed here.^17,18^

## Conclusions

In conclusion, we have characterized the low-temperature magnetic behavior of a molecular Co^2+^ complex which crystallizes as a layered material. EPR measurements suggest that the gradual depopulation of the *J* = 3/2 orbital triplet level in favor of the *J* = 1/2 level occurs such that at 20 K, the spectra can be modeled as an *S*_eff_ = 1/2 state. Fits to the magnetic susceptibility using a two-level model indicate a gap in the energy of these two states of approximately 88 K and a much smaller Weiss constant than previously suggested for this compound on the basis of Curie–Weiss fits. While the material no longer appears to feature significant magnetic frustration, there is evidence in the bulk magnetic properties and EPR measurements of ferromagnetic interactions at low temperatures that are not accounted for by this two-level model. Heat capacity measurements suggest the emergence of magnetic order around 246 mK, with neutron diffraction finding this to be to a collinear ferromagnetic structure, with both the entropy change associated with this magnetic order and ordered moment in the magnetic structure broadly consistent with the Co^2+^ cations adopting a *J* = 1/2 state. This behavior emphasizes the need for careful analysis of Co^2+^ magnets to fully interpret their behavior.

## Experimental Section

### Materials

All reagents and solvents employed were obtained from commercial sources and were used without further purification unless otherwise stated.

### Synthesis

#### CoHyd_2_Cl_4_ Synthesis

**CoHyd**_**2**_**Cl**_**4**_ was synthesized using a modification of a previously published procedure.^34^ CoCl_2_·6H_2_O (340 mg, 1.43 mmol) was dissolved in 10 mL of a 20 mL MeOH:H_2_O solution (MeOH: 9.1 mL, H_2_O: 10.9 mL), while hydrazine hydrochloride (196 mg, 2.86 mmol) was dissolved in the remaining 10 mL. The solutions were then added together and stirred until all residual solids had dissolved with the resulting mixture transferred to a Schlenk tube and held under a positive pressure of N_2_ gas until the solvent evaporated to yield the product as a dark pink powder (380 mg, 1.25 mmol, 88%).

Deuterated **CoHyd**_**2**_**Cl**_**4**_ was obtained using the above procedure with the substitution of the MeOH and H_2_O solvents for MeOD and D_2_O. Once the dried powder had been obtained, the sample was handled and stored under N_2_ at all times prior to measurement to prevent exchange of deuterons with atmospheric water.

### Physical Characterization and Instrumentation

#### Powder X-ray Diffraction

X-ray powder diffraction patterns were obtained using a Rigaku MiniFlex diffractometer equipped with a D/teX Ultra silicon strip detector and employing Cu K_α_ (λ = 1.5406 Å) radiation.

#### SQUID Heat Capacity and Magnetometry

The heat capacity measurements were performed using a DynaCool Physical Property Measurement System from Quantum Design capable of measuring heat capacity (*C*_*p*_) in the range of 50 mK to 4 K. Preceding this, a background (addenda) measurement was performed over the same temperature range taking into account the thermal Apiezon N grease used to secure the sample to the platform and ensure a good thermal contact. The measurement itself then mounted the sample of 2.48 mg onto the platform. Magnetic susceptibility measurements were performed using a SQUID vibrating sample magnetometer, also from Quantum Design, measuring in the range of 1.8 K – 400 K, ± 7 T.

#### Neutron Powder Diffraction

Neutron powder diffraction patterns were obtained using the high-resolution time-of-flight (TOF) WISH diffractometer at the ISIS Neutron and Muon Source, Rutherford Appleton Laboratory.^45^ Measurements were carried out between 87 mK and 20 K with the sample loaded into an 8 mm copper can and cooled in a dilution refrigerator inside the standard cryostat. Rietveld refinements of the diffraction data were carried out using the FullProf software package^49^ with the copper sample can environment peaks fitted using the Le Bail method and the peak profiles fitted using a convolution of a back-to-back exponential and pseudo-Voigt TOF functions.

#### Electron Paramagnetic Resonance

Variable-temperature, continuous-wave X-band (ca. 9 GHz) and Q-band (ca. 34 GHz) EPR measurements were carried out on two Bruker Elexsys E580 platforms. X-band measurements were performed by using a Bruker SuperX FT-EPR microwave bridge mated to a Bruker ER4118X-MD5 Flexline resonator. Q-band was performed using a Bruker SuperQ-FT bridge mated to a Bruker ER5106-QT Flexline resonator. Cryogenic temperatures were achieved utilizing identical Cryogenic Ltd. CF-VTC for EPR (closed-cycle cryostats) installed on each platform. Q-Band measurements were subsequently verified using the same resonator on a Bruker EMXPlus platform equipped with a ColdEdge Stinger, closed-cycle cryocooler. Field offset correction was carried out against the Bruker strong pitch calibration standard sample (*g* = 2.0028), and spectral simulations were carried out using the EasySpin simulation package.^50^ Typical measurement parameters used were microwave power: 2 mW, modulation amplitude: 4 G, and sweep times: 120 s. Samples were measured as finely ground powders within quartz EPR tubes (QSIL GmbH).

## Supporting Information Available

Additional fitted powder X-ray and neutron diffraction pattern, crystallographic parameters, EPR spectra, magnetic property, and heat capacity measurements (PDF)
Magnetic crystallographic information file of the magnetic structure determined in this study (MCIF)

## Supplementary Material

cm4c00596_si_001.pdf

cm4c00596_si_002.mcif
