# Supplementary material for: Low-Temperature Ferromagnetic Order in a Two-Level Layered Co2+ Material
Source: Chem Mater. 2024 Aug 9;36(17):8208–16. doi: 10.1021/acs.chemmater.4c00596 (PMC11393796; doi:10.1021/acs.chemmater.4c00596)
Supplement: Supplementary file 1 — cm4c00596_si_001.pdf [file cm4c00596_si_001.pdf]

Low Temperature Ferromagnetic Order in a Two-level Layered Co^{2+} Material

Patrick W. Doheny,[a] Gavin B. G. Stenning,[b] Adam Brookfield,[c] Fabio Orlandi,[b] David Collison,[c] Pascal Manuel,[b] Sam T. Carr[d] and Paul J. Saines*[a]

[a] School of Chemistry and Forensic Science, Ingram Building, University of Kent, Canterbury, CT2 7NH, United Kingdom

[b] ISIS Neutron and Muon Source, Rutherford Appleton Laboratory, Chilton, Didcot, OX11 0QX, United Kingdom

[c] Department of Chemistry and Photon Science Institute, EPSRC National Research Facility for Electron Paramagnetic Resonance Spectroscopy, The University of Manchester, Manchester M13 9PL, UK.

[d] School of Physics and Astronomy, Ingram Building, University of Kent, Canterbury, CT2 7NH, United Kingdom

* Corresponding author's email: P.Saines@kent.ac.uk

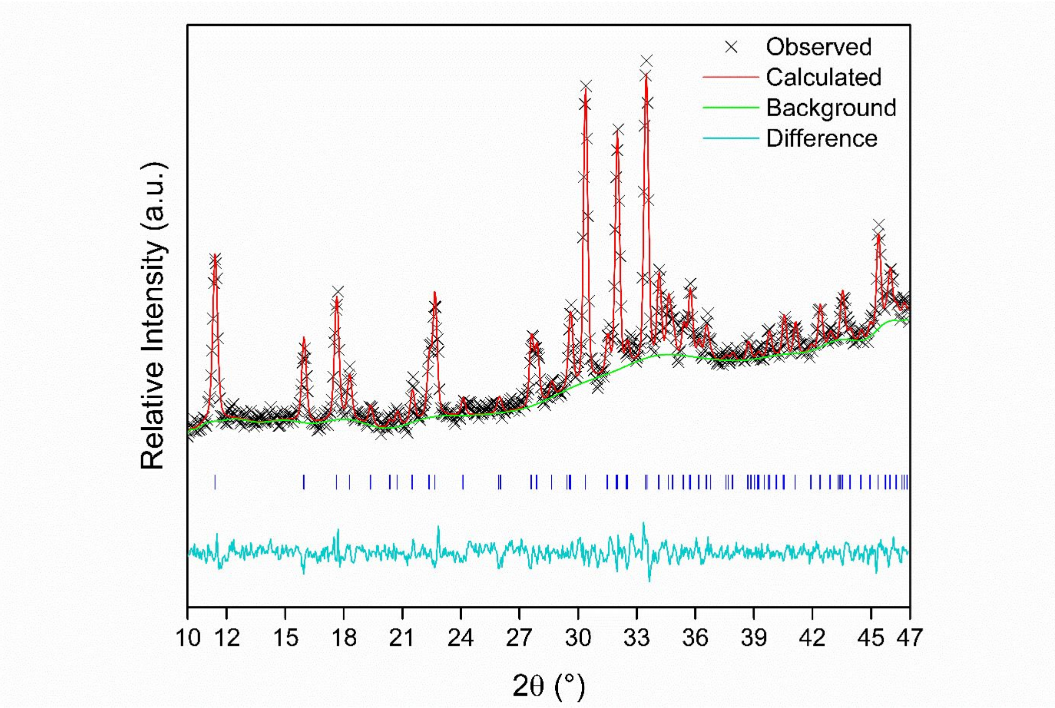

Figure S1. Le Bail fit of the deuterated **$CoHyd_2Cl_4$** X-ray powder diffraction pattern where a = 7.9989(19) Å, b = 5.7187(9) Å, c = 11.3761(3) Å, β = 97.639(9) ° and V = 515.77(2) $Å^3$. R_p = 1.80%, R_{wp} = 2.31% and reduced χ^2 = 2.11.

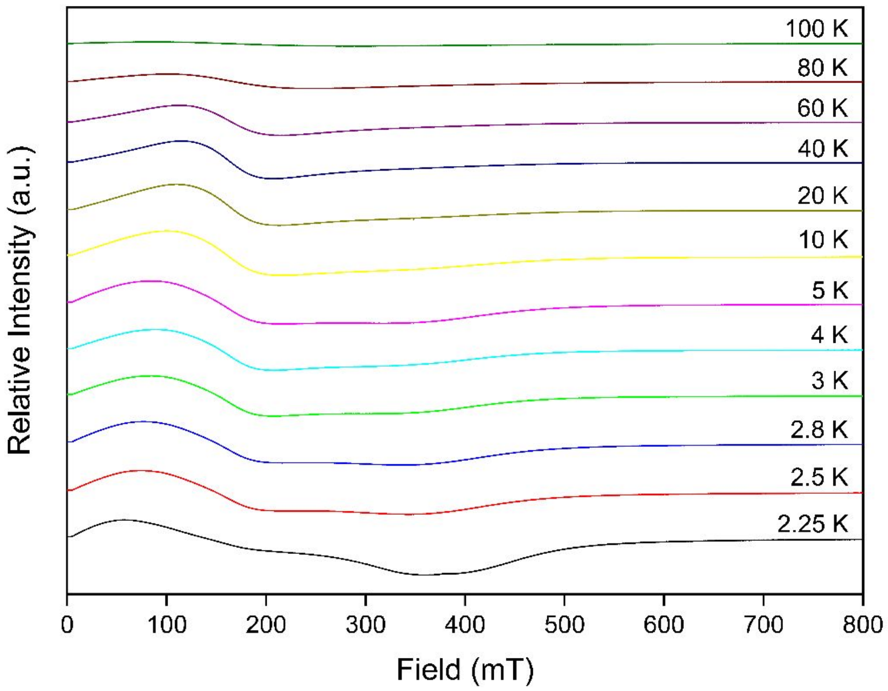

Figure S2. Variable temperature X-band EPR spectroscopy of the **$CoHyd_2Cl_4$** material carried out over a 2.25-200 K temperature range. The band envelopes of the unresolved g-anisotropy correspond to the range of g-values determined from the Q-band data.

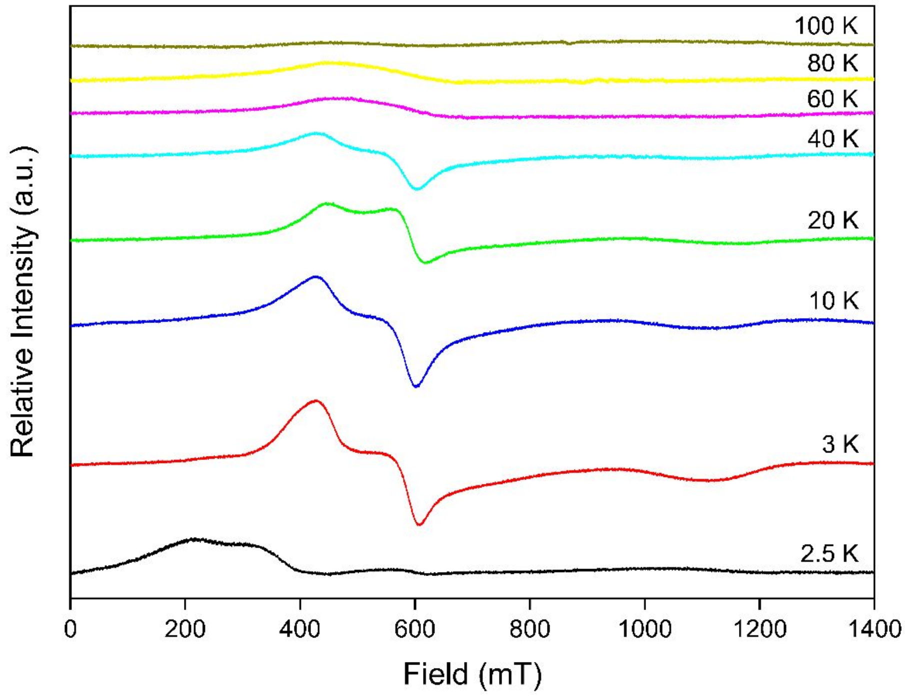

Figure S3. Variable temperature Q-band EPR spectroscopy of the **CoHyd$_2$Cl$_4$** material carried out over a 2.5-100 K temperature range.

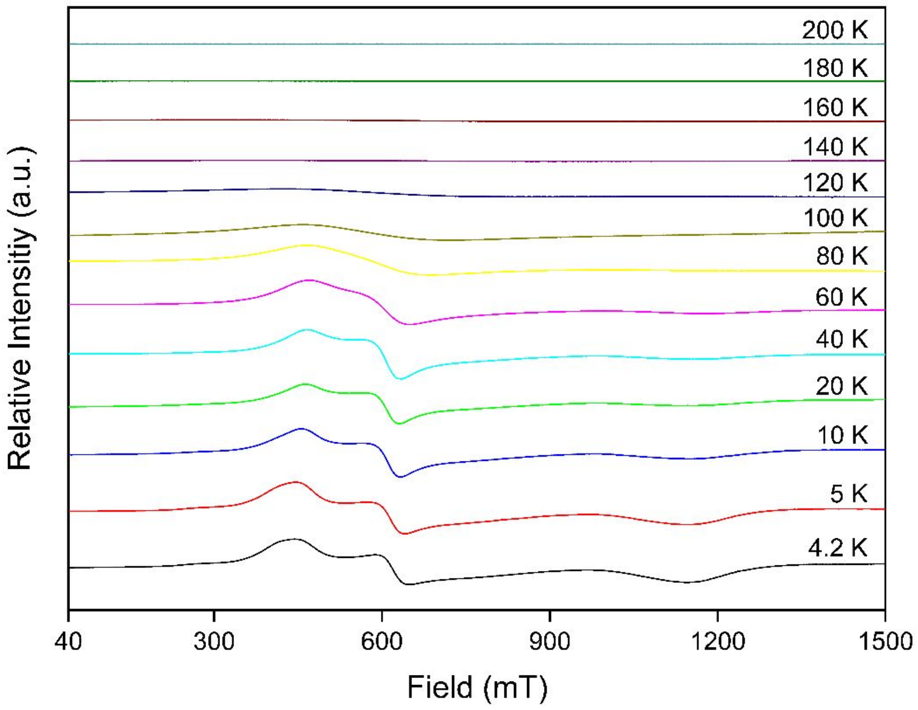

Figure S4. Variable temperature Q-band EPR spectroscopy of the **CoHyd$_2$Cl$_4$** material carried out over a 4.2-200 K temperature range.

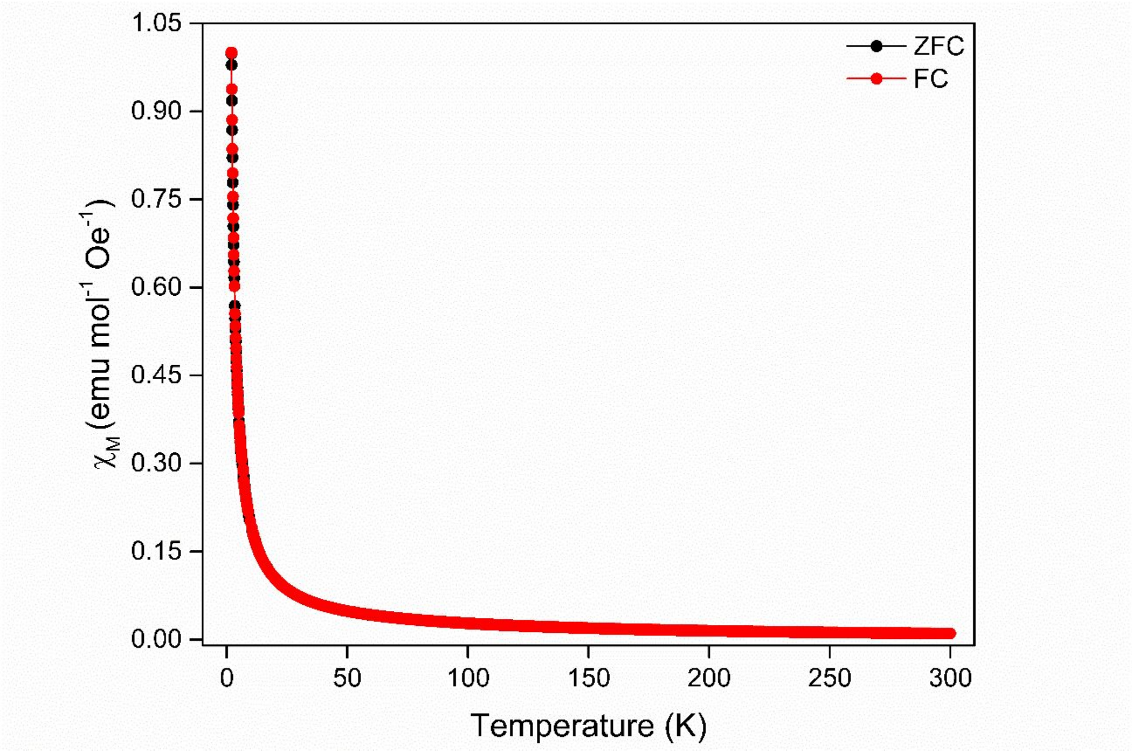

Figure S5. ZFC and FC magnetic susceptibility of **$CoHyd_2Cl_4$** in a 1000 Oe field over a 1.8-300 K temperature range.

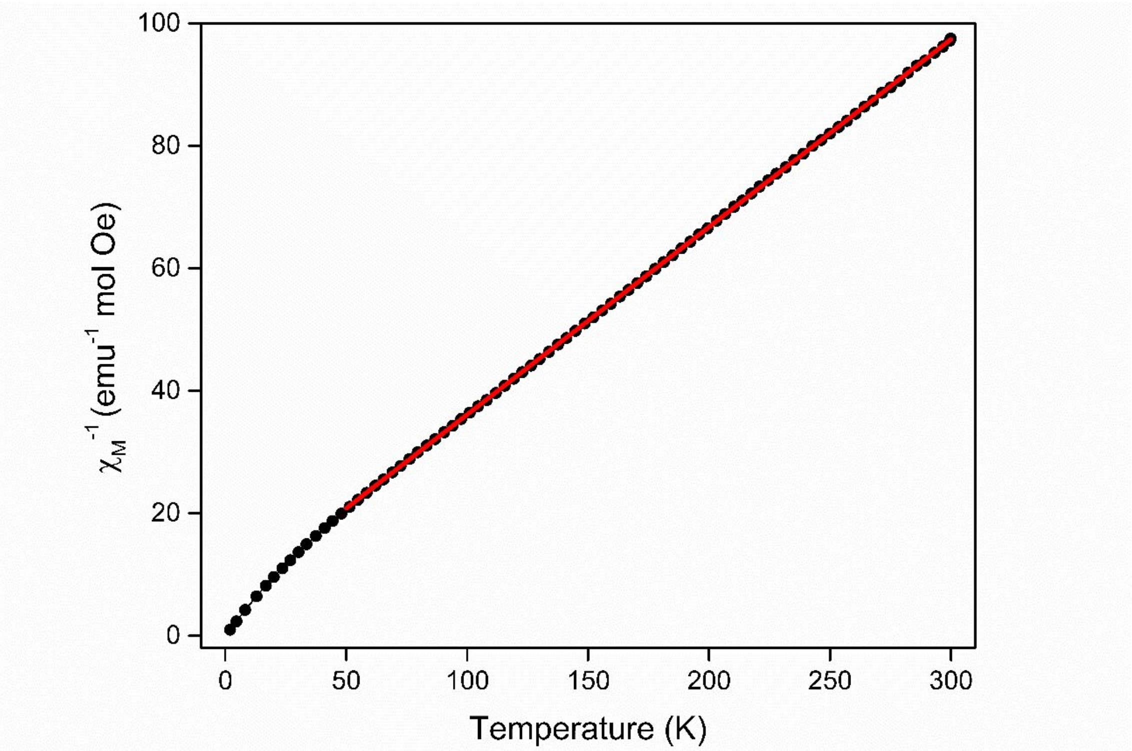

Figure S6. Curie-Weiss fits of the inverse susceptibility of **$CoHyd_2Cl_4$** over a 50-300 K (red), temperature range.

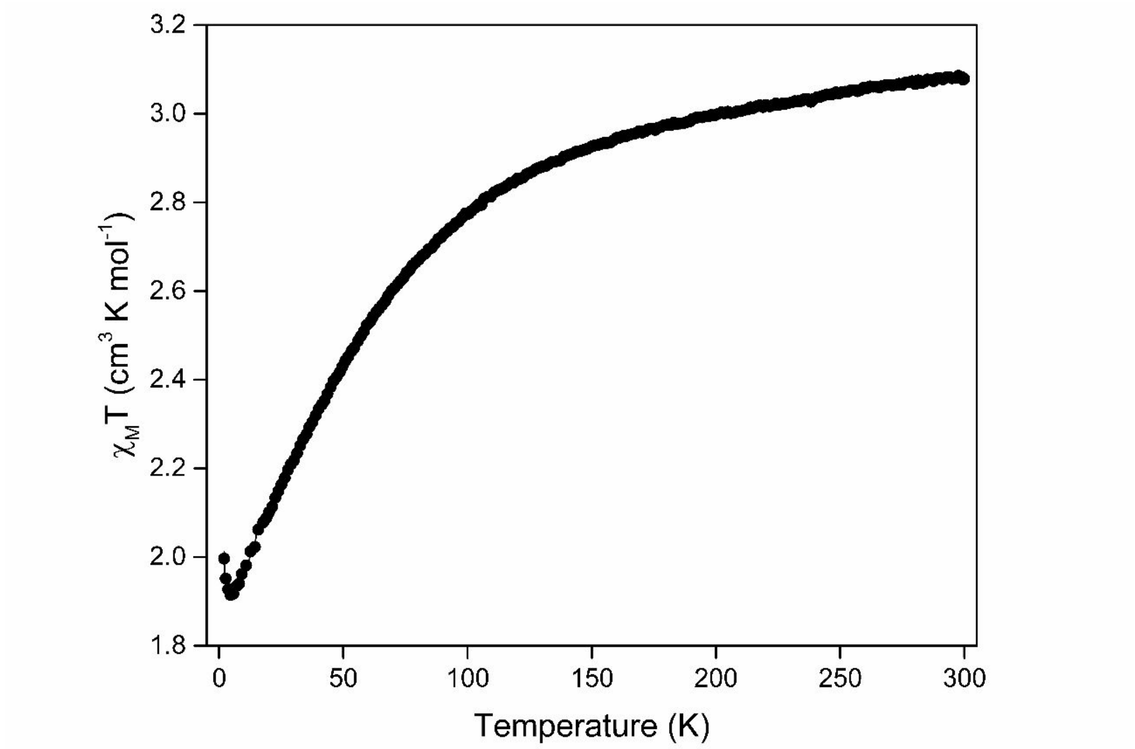

Figure S7. $\chi_M T$ data of **CoHyd$_2$Cl$_4$** over a 1.8-300 K temperature range.

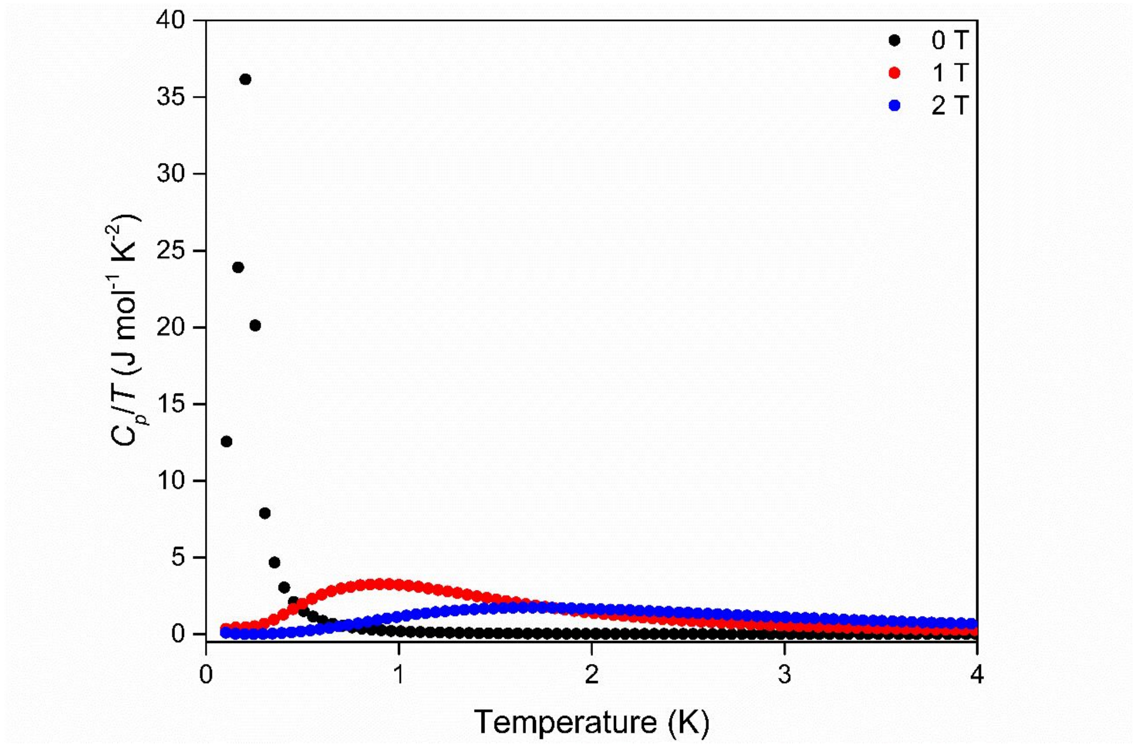

Figure S8. C_p/T data for **CoHyd$_2$Cl$_4$** from 0.1-4.0 K under applied fields of 0-2 T.

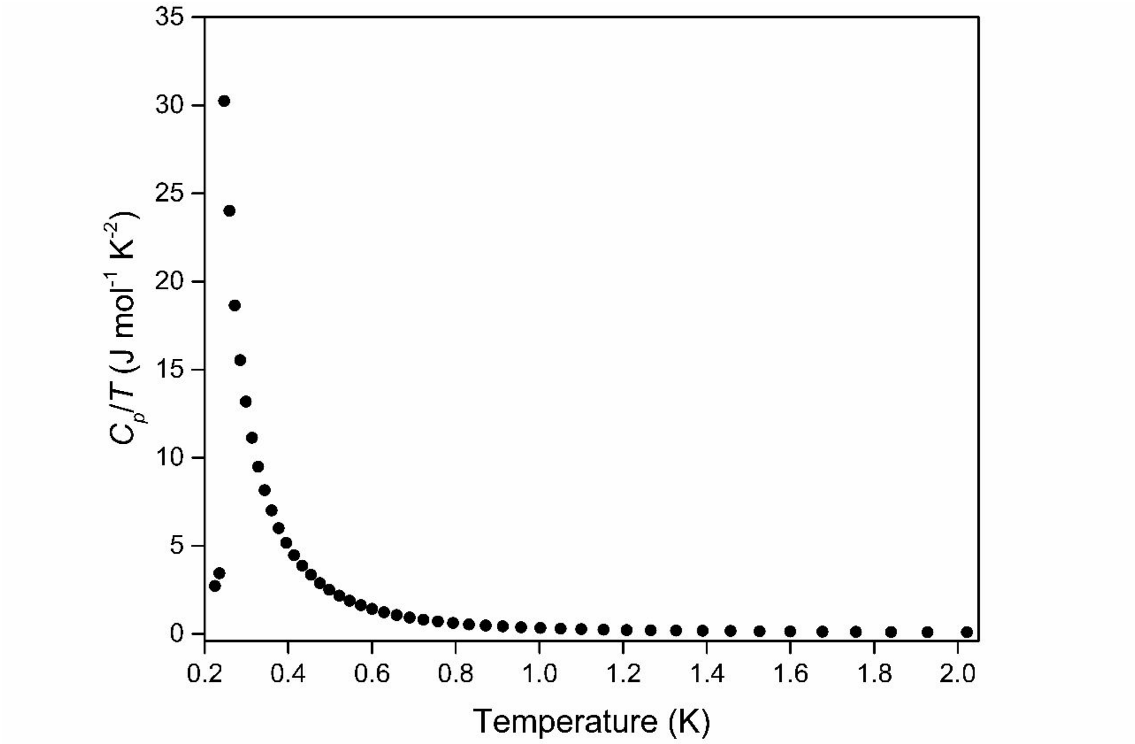

Figure S9. C_p/T data for $\mathbf{CoHyd_2Cl_4}$ from 0.224-2.2 K under 0 T.

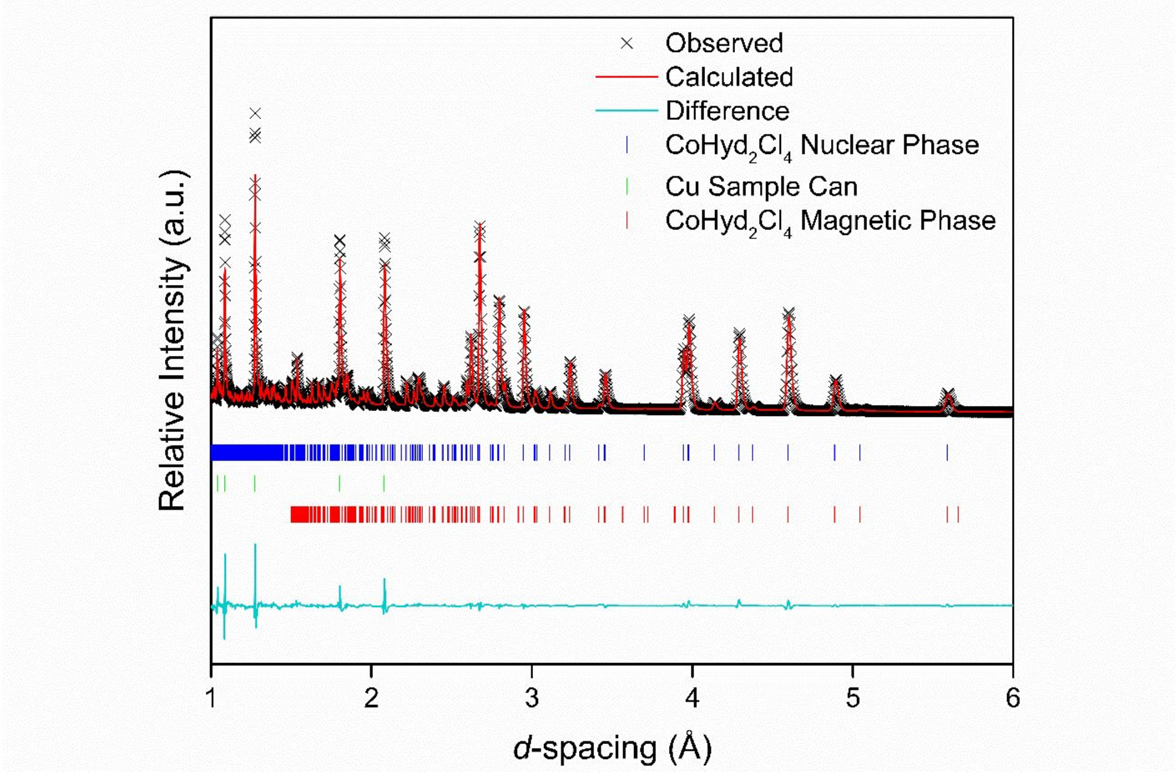

Figure S10. Rietveld refinement profile of the $\mathbf{CoHyd_2Cl_4}$ neutron powder diffraction data obtained at 87 mK from WISH detector banks 4 and 7, with average 2θ=121.66° where a = 7.9605(4) Å, b = 5.6554(3) Å, c = 11.2818(6) Å, β = 97.926(3) ° and V = 503.05(5). R_p = 12.2% and R_{wp} = 9.66%.

Table S1. Atomic fractional coordinates, thermal parameters and site occupancy factors of the **$CoHyd_2Cl_4$** nuclear unit cell at 87 mK. The displacement parameters of the distinct functional groups in the hydrazinium molecules, chloride ions and water molecules were fixed to be equal. The occupancies of the deuterium sites were refined to establish the extent of deuteration in the compound with the values found consistent with all sites having at least 90 % D.

Atom	x	y	z	B_{iso}	SOF
Co1	0.5000	0.5000	0.5000	-0.06(8)	0.50000
Cl1	0.4921(9)	0.8071(10)	0.3536(7)	0.4(2)	1.00000
Cl2	0.9633(8)	0.6796(12)	0.3558(7)	0.4(2)	1.00000
O1	0.306(2)	0.688(2)	0.5746(13)	0.4(3)	1.00000
D1A	0.3592(14)	0.751(2)	0.6583(10)	1.2(2)	0.944(12)
D1B	0.220(2)	0.581(2)	0.6046(11)	1.2(2)	0.944(12)
N1	0.7083(9)	0.654(2)	0.6210(8)	0.3(2)	1.00000
D1C	0.672(2)	0.705(2)	0.7016(12)	0.6(3)	0.87(2)
D1D	0.7982(15)	0.529(2)	0.6347(11)	0.6(3)	0.87(2)
N2	0.8032(12)	0.858(2)	0.5801(8)	1.1(2)	1.00000
D2A	0.8569(14)	0.817(2)	0.5033(11)	0.9(3)	0.88(2)
D2B	0.890(2)	0.919(2)	0.6494(11)	0.9(3)	0.88(2)
D2C	0.7152(18)	0.988(2)	0.5573(9)	0.9(3)	0.88(2)

Table S2. Bond distances of **$CoHyd_2Cl_4$** determined from Rietveld refinement at 87 mK.

Atom 1	Atom 2	Bond Distance (Å)
Co1	Cl1	2.392(7)
Co1	O1	2.141(14)
O1	D1A	1.043(18)
O1	D2B	1.007(18)
Co1	N1	2.179(8)
N1	D1C	1.033(17)
N1	D1D	1.004(14)
N1	N2	1.487(14)
N2	D2A	1.044(17)
N2	D2B	1.024(16)
N2	D2C	1.029(15)

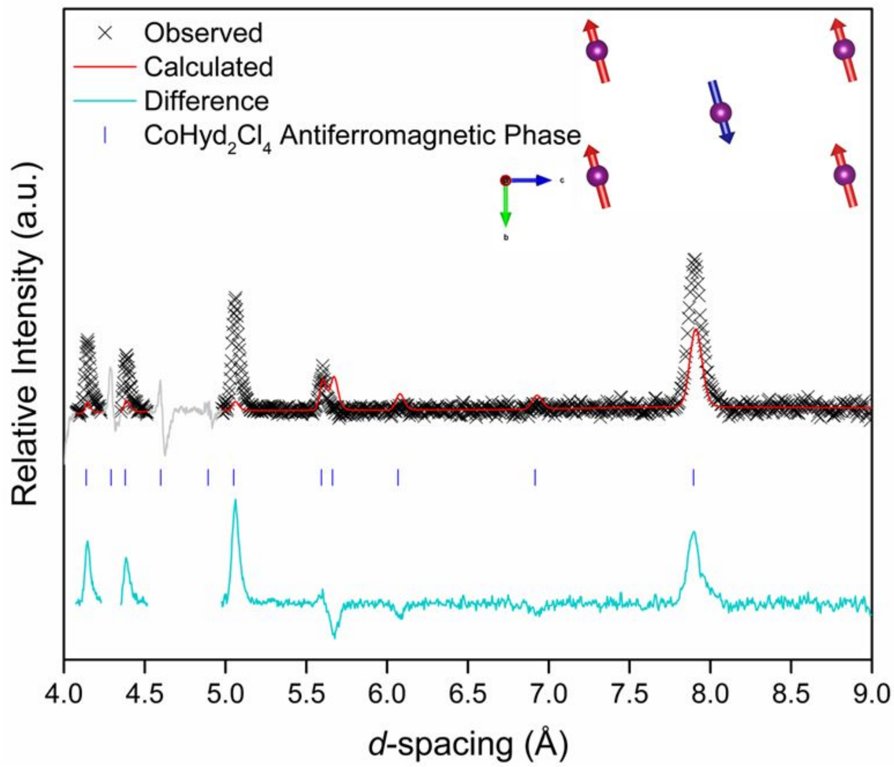

Figure S11. Magnetic Rietveld refinement profile of the antiferromagnetic magnetic structure to neutron powder diffraction data at 87 mK from which data collected at 20 K have been subtracted. Note the antiferromagnetic magnetic structure is unable to reproduce the intensity of the magnetic Bragg peaks successfully and was discarded in favour of the ferromagnetic model. The grey regions in these data were excluded from the refinement as they correspond to strong nuclear reflections. Insert: The antiferromagnetic chain model used in the refinement.

Table S3. Magnetic atom coordinates and moment for the ferromagnetic $CoHyd_2Cl_4$ magnetic structure in the $P2_1'/c'$ magnetic space group.

Atom	x	y	z	Moment (μ_B)
Co1	0.5	0	0	1.69(2)

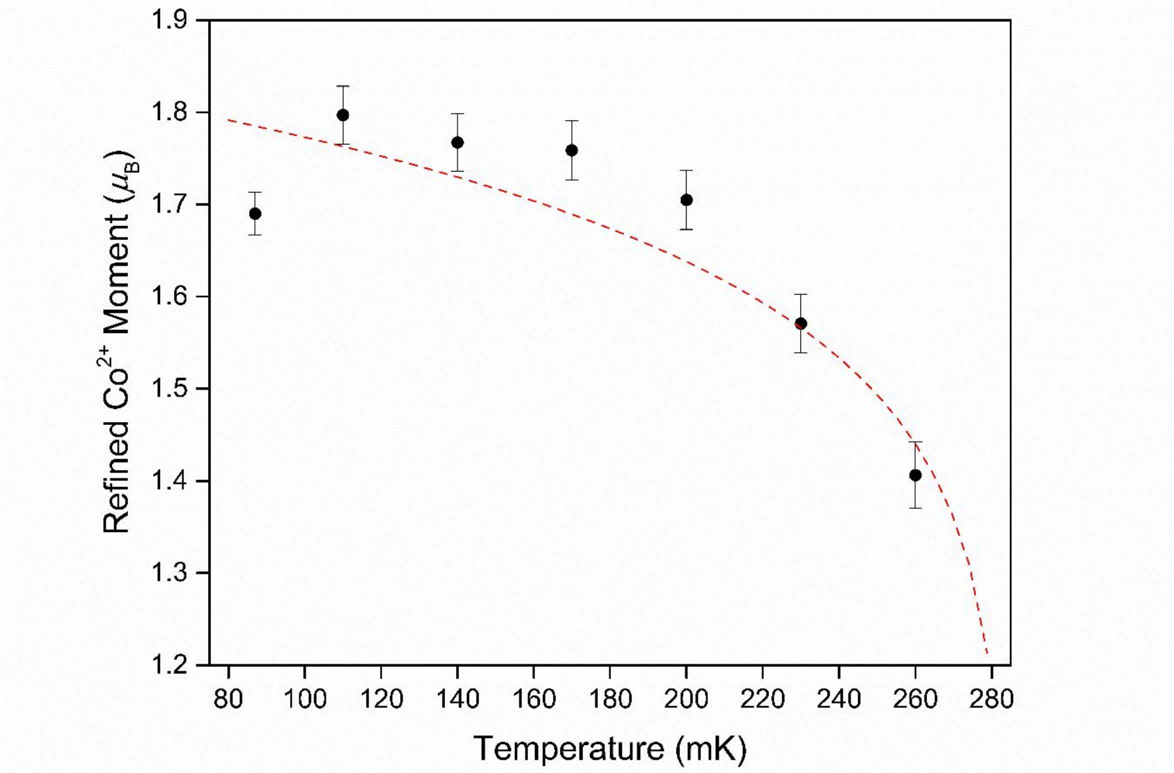

Figure S12. Magnetic moments obtained from neutron powder diffraction data of **$CoHyd_2Cl_4$** from 87-260 mK on WISH detector banks 2 and 9 with an average 2θ of 58.33°. The red dashed line is a guide to the eye for the evolution of the magnetic moment.
